# Allicin Facilitates Airway Surface Liquid Hydration by Activation of CFTR

**DOI:** 10.3389/fphar.2022.890284

**Published:** 2022-06-15

**Authors:** Zhuo-Er Qiu, Jian-Bang Xu, Lei Chen, Ze-Xin Huang, Tian-Lun Lei, Zi-Yang Huang, Xiao-Chun Hou, Hai-Long Yang, Qin-Hua Lin, Yun-Xin Zhu, Lei Zhao, Wen-Liang Zhou, Yi-Lin Zhang

**Affiliations:** ^1^ School of Life Sciences, Sun Yat-sen University, Guangzhou, China; ^2^ State Key Laboratory of Respiratory Disease, National Clinical Research Center for Respiratory Disease, Guangzhou Institute of Respiratory Disease, The First Affiliated Hospital of Guangzhou Medical University, Guangzhou Medical University, Guangzhou, China; ^3^ Department of Physiology, School of Basic Medical Sciences, Guangzhou Medical University, Guangzhou, China

**Keywords:** allicin, airway epithelium, Cl^−^ secretion, CFTR (cystic fibrosis transmembrane conductance regulator), airway surface liquid (ASL)

## Abstract

Airway epithelium plays critical roles in regulating airway surface liquid (ASL), the alteration of which causes mucus stasis symptoms. Allicin is a compound released from garlic and harbors the capacity of lung-protection. However, the potential regulatory effects of allicin on airway epithelium remain elusive. This study aimed to investigate the effects of allicin on ion transport across airway epithelium and evaluate its potential as an expectorant. Application of allicin induced Cl^−^ secretion across airway epithelium in a concentration-dependent manner. Blockade of cystic fibrosis transmembrane conductance regulator (CFTR) or inhibition of adenylate cyclase-cAMP signaling pathway attenuated allicin-induced Cl^−^ secretion in airway epithelial cells. The *in vivo* study showed that inhaled allicin significantly increased the ASL secretion in mice. These results suggest that allicin induces Cl^−^ and fluid secretion across airway epithelium via activation of CFTR, which might provide therapeutic strategies for the treatment of chronic pulmonary diseases associated with ASL dehydration.

## Introduction

As the first line of defense against external invasion, airway epithelium acts as a barrier to the particles and pathogens deposited in the airways (Tam et al., 2011). The luminal surface of airway epithelium is covered by airway surface liquid (ASL), which arises mainly from submucosal gland secretions and transepithelial electrolyte and water transport (Widdicombe, 2002) (Tam et al., 2011). ASL comprises a mucus layer and a thin layer of fluid termed periciliary layer (PCL) that surrounds the cilia. The mucus traps inhaled particles for removal by mucociliary clearance, while PCL keeps the mucins at a sufficient distance from the ciliated epithelial cells for optimal ciliary beating (Toosi, 2014) (Tarran et al., 2006). However, when the ASL homeostasis is impaired, it causes dehydration of the airway surfaces, hyperconcentrated mucus, failure in mucus transport, and mucus adhesion to airway surfaces (Boucher, 2007b). Mucus stasis then contributes to the airflow obstruction, persistent and progressive infection, and inflammatory characteristics of chronic obstructive lung diseases including cystic fibrosis (CF), chronic obstructive pulmonary disease (COPD) and chronic bronchitis (Boucher, 2007a) (Boucher, 2004) (Kesimer et al., 2018) (Evans and Koo, 2009). ASL hydration and mucociliary transport are regulated by transepithelial ion transport processes, mainly luminal Cl^−^ secretion and Na^+^ absorption (Lazarowski and Boucher, 2021). As the major apically located anion channel, cystic fibrosis transmembrane conductance regulator (CFTR) plays crucial roles in the regulation of electrolytes and fluid secretion across the airway epithelium (Boucher, 2019). Mutations in the CFTR gene that result in abnormal Cl^−^ secretion are also associated with enhanced Na^+^ absorption (Gentzsch et al., 2010), leading to ASL volume depletion (Tyrrell et al., 2015) (Cantin, 2016). Therefore, drugs with regulatory effects on CFTR-mediated Cl^−^ secretion may relieve symptoms of the airway diseases associated with ASL dehydration to some extent.

Allicin (IUPAC name: 3-prop-2-enylsulfinylsulfanylprop-1-ene) is a diallyl thiosulfonate generated from alliin in garlic (*Allium sativum* L.) via interaction with alliinase (Stoll and Seebeck, 1947). As the major active components of garlic, allicin harbors the property of anti-tumor, anti-diabetic, anti-atherosclerotic, lung-protective, liver-protective and cardio-protective bioactivities (Rahman, 2007) (Dixit and Chaudhary, 2014) (Mandal et al., 2019). Previous studies showed that allicin has a bactericidal function against pulmonary pathogens such as *pseudomonas*, *streptococcus* and *staphylococcus* (Reiter et al., 2017) (Cañizares et al., 2004). However, the regulatory effects of allicin on airway epithelium remains unclear. Allicin can oxidize sulfhydryl groups and cysteine residues on proteins, thereby changing the redox state of cells and protein structure (Gruhlke and Slusarenko, 2012). In addition, allicin reportedly increased intracellular cAMP content and regulated vascular relaxation through endothelium-derived hyperpolarizing factor pathway in vascular endothelial cells (Cui et al., 2020). Given that CFTR is activated by increased intracellular cAMP levels, it is plausible that allicin may modulate ASL volume homeostasis by facilitating Cl^−^ secretion through activating CFTR. Therefore, this study aims to investigate mechanisms underlying the regulatory effect of allicin on transepithelial ion transport in airway epithelium and evaluate its potential as an expectorant.

## Materials and Methods

### Reagent

Minimum essential medium, fetal bovine serum, penicillin/streptomycin and trypsin were purchased from Gibco (Grand Island, NY, United States). Allicin was provided by Ailexin (Urumuqi, China). Amiloride, CFTRinh-172, DIDS, forskolin (FSK), isobutylmethylxanthine (IBMX) were purchased from Sigma-Aldrich (Missouri, United States). SQ22536 was purchased from MedChemExpress (New Jersey, United States). NaCl, KCl, MgSO_4_, NaHCO_3_, KH_2_PO_4_, CaCl_2_, glucose, and gluconate were purchased from Guangzhou Chemical Pharmaceutical Factory (Guangzhou, China). HEPES was purchased from Mbchem Technology (Guangzhou, China). The direct cAMP enzyme immunoassay kit (KGE002B) was purchased from R&D Systems (Minneapolis, MN, United States). The bicinchoninic acid protein assay kit (kw0014) was purchased from KWBIO (Beijing, China).

### Animals

Kunming mice weighing 25–30 g were purchased from the Laboratory Animal Center of Sun Yat-sen University (Guangzhou, China). The mice were housed in specific pathogen-free conditions with a constant room temperature of 20°C and a 12 L:12 D photoperiod with food and water allowed *ad libitum*. All procedures were approved by the Institutional Animal Care and Use Committee (IACUC), Sun Yat-sen University (Guangzhou, China).

### Cell Culture

Human bronchial epithelial cell line 16HBE14o- and CF human bronchial epithelial cell line CFBE41o- were obtained as a kind gift from Prof. Wing-Hung Ko (School of Biomedical Sciences, The Chinese University of Hong Kong, Hong Kong, China). The cells were cultured in minimum essential medium supplemented with 10% (v/v) fetal bovine serum and 1% (v/v) penicillin−streptomycin at 37°C with 5% CO_2_ in a humidified atmosphere.

### Short Circuit Current (*I*_SC_) Measurements

The measurement of *I*_SC_ was performed following a modified procedure as previously described (Zhang et al., 2018) (Cottrill et al., 2021). In brief, 16HBE14o- or CFBE41o- cell monolayers grown on Millipore filter membranes (Millipore) with a pore diameter of 0.45 μm were clamped vertically between the two halves of an Ussing chamber (EM-CSYS-2 Ussing Chamber Systems, Physiologic Instruments, San Diego, United States). The confluent monolayer of cells was bathed in Krebs-Henseleit (K-H) solution (115 mM NaCl, 5 mM KCl, 1 mM MgCl_2_, 2 mM CaCl_2_, 10 mM glucose, 25 mM NaHCO_3_) at the basolateral side and with reduced Cl^−^ concentration (115 mM Na-gluconate, 5 mM KCl, 1 mM MgCl_2_, 4 mM CaCl_2_, 10 mM glucose, 25 mM NaHCO_3_) at the apical side to generate a basolateral to apical Cl^−^ gradient. The solution was gassed with 95% O_2_/5% CO_2_ to obtain a constant pH of 7.4 and maintained at 37°C during the experiments. The epithelium exhibited a basal transepithelial potential difference (PD), which was measured by Ag/AgCl electrodes with KCl/agar bridges connected to the voltage-clamp amplifier (VCC MC6, Physiologic Instruments, San Diego, United States). For *I*_SC_ measurement, the transepithelial PD of the confluent monolayer was clamped at 0 mV. The change of *I*_SC_ (Δ*I*_SC_), defined as the difference between the value at baseline and that at a peak following the stimulation, was synchronously displayed via a signal collection and analysis system (BL-420E+ system, Chengdu Technology & Market Co. Ltd., Chengdu, China) and normalized by the unit area of the preparation (ΔμA/cm^2^). At the beginning and the end of each experiment, 1 mV pulse was applied and the current change in response was used to estimate transepithelial resistance according to Ohm’s law. In the ion substitution experiments, HCO_3_^−^ was substituted by HEPES, and Cl^−^ was substituted by gluconate. HCO_3_^−^-free solution was ventilated in 100% O_2_.

### Intracellular cAMP Analysis

Intracellular cAMP content was measured using a direct cAMP enzyme immunoassay kit (R&D Systems, KGE002B, Minneapolis, MN, United States) according to the instructions. The assay was based on the competitive binding technique by using monoclonal antibodies specific for cAMP. The total protein content in the lysate was measured using the bicinchoninic acid protein assay kit (KWBIO, kw0014, Beijing, China).

### Evaluation of Pro-secretory Activity of Allicin

The potent pro-secretory activity of allicin was evaluated by the phenol red secretion assay as previously described (Menezes et al., 2019). Briefly, the mice were treated with saline, allicin (100 μM, 200 μM, and 400 μM as low-dose, middle-dose and high-dose separately) or salbutamol (1 mg/ml) by aerosol for 30 min lasting for four consecutive days. The mice were intraperitoneally injected with 5% (w/v) phenol red dissolved in saline (50 μL/10 g body weight) or an aliquot of saline as the blank control 30 min after the last nebulization. Then, the mice were euthanized by cervical dislocation 30 min after the injection of phenol red and bronchoalveolar fluid (BALF) samples were collected. The BALF samples were centrifuged at 650 × *g* for 10 min and alkalinized with NaOH (0.1 mM). Then, the absorbance was measured at 565 nm wavelength in a spectrophotometer. The standard curve of phenol red was prepared with various concentrations of phenol red solutions (0, 0.5, 1, 5, 10, 50, 100 ng/ml). The calibration curve could be obtained by fitting the phenol red concentration corresponding to absorbance with the linear function “y = kx + b”. The concentration of phenol red (ng/ml) in BALF samples was then calculated.

### Data Analysis and Statistics

All data were presented as the mean ± standard deviation (S.D.) with distinct dots for each measurement. The Student’s *t*-test (two-tailed) was used to compare the differences between two groups. For three or more groups, data were analyzed with one-way analysis of variance followed by Tukey’s multiple comparison tests (GraphPad Software, Inc, San Diego, CA, United States). Statistically significant differences between groups were defined as *p* < 0.05.

## Result

### Allicin Stimulated an Increased *I*_SC_ Response in Human Airway Epithelium

**FIGURE 1:**
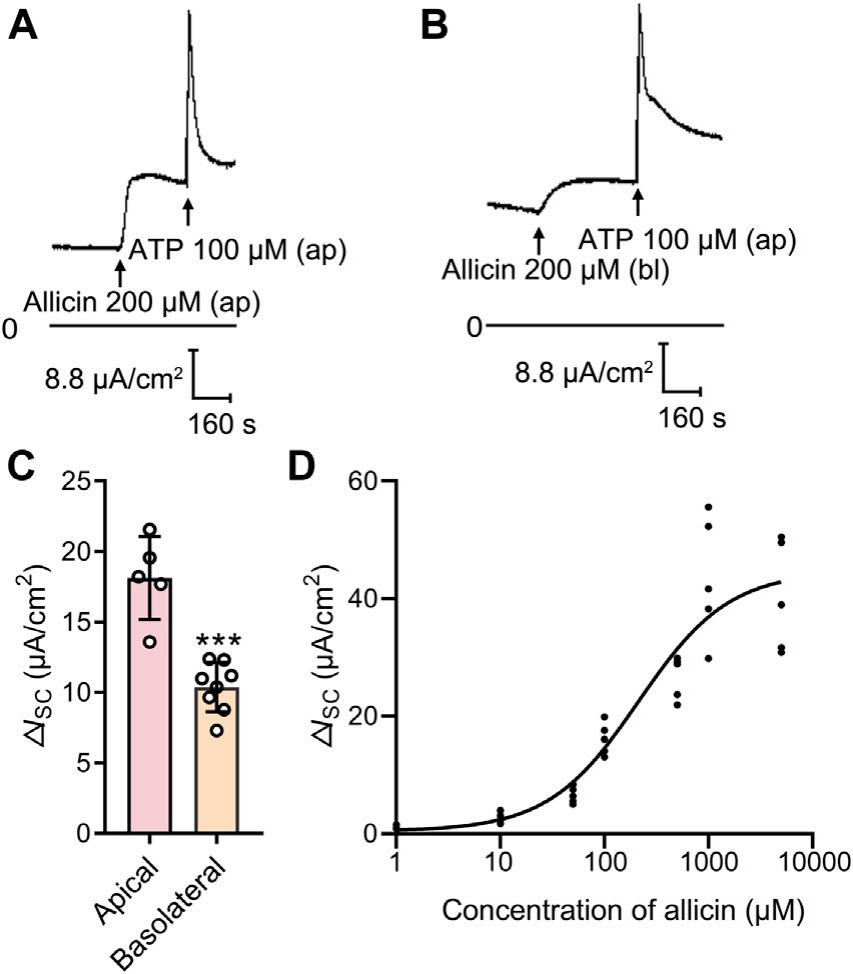
Allicin dose-dependently stimulated an increased short-circuit current (*I*_SC_) response in human airway epithelium. **(A,B)** Representative traces of the *I*_SC_ responses induced by **(A)** apical or **(B)** basolateral application of allicin (200 μM) in 16HBE14o- cells, with **(C)** the corresponding statistical analysis. Each column and error bar indicate the mean ± SD. ^***^ *p* < 0.001 compared with the apical applied group. Ap, apical application; Bl, basolateral application. **(D)** Dose-response curve of the apical application allicin-stimulated *I*_SC_ response. The concentrations of allicin were 1, 10, 50, 100, 500, 1,000 and 5,000 μM.

To investigate the effect of allicin on ion transport in airway epithelium, the *I*_SC_ was measured by using the Ussing chamber technique. In 16HBE14o- cell monolayers, the mean basal *I*_SC_ was 28.38 ± 8.32 μA/cm^2^ (*n* = 37), with the transepithelial resistance of 2,837 ± 462.7 Ω cm^2^ (*n* = 16) and the mean transepithelial PD of −6.55 ± 0.85 mV (*n* = 17). Apical application of allicin (200 μM) triggered a sustained increase in *I*_SC_ (Figure 1A) in a concentration-dependent manner (Figure 1D), with a half-maximal effective concentration at 213.6 μM. However, basolateral application of allicin induced a relatively blunted response (Figures 1B, C). Hence, 200 μM allicin was apically applied in the subsequent experiments to investigate its biological effect on airway epithelial cells.

### Allicin Induced Cl^−^ Secretion in Human Airway Epithelium

**FIGURE 2:**
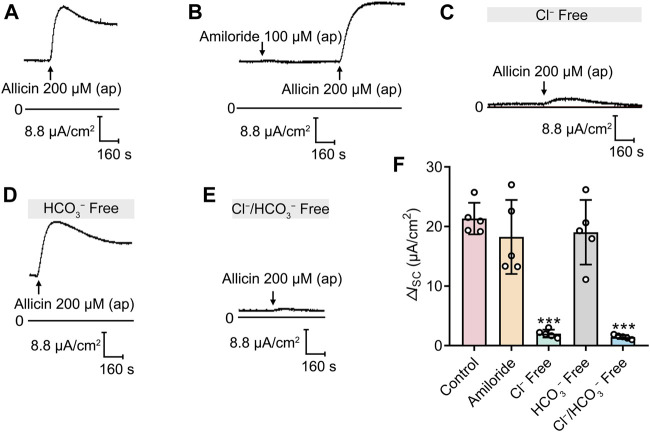
The short-circuit current (*I*_SC_) response stimulated by allicin was Cl^−^ dependent. **(A–E)** Representative traces of the *I*_SC_ responses induced by the apical application of allicin (200 μM) in 16HBE14o- cells in **(A)** normal solution without or **(B)** with pretreatment of amiloride (100 μM), or **(C)** in Cl^−^ free, **(D)** HCO_3_^−^ free, or **(E)** Cl^−^/HCO_3_^−^ both free solution, with **(F)** the corresponding statistical analysis. Each column and error bar indicate the mean ± SD. ^***^ *p* < 0.001 compared with the control.

To determine the ion composition involved in the allicin-induced *I*_SC_ response, a series of channel blocker tests and ion substitution experiments were carried out. As the epithelial Na^+^ channel (ENaC) plays an important role in pulmonary fluid regulation (Matalon et al., 2015), we initially investigated whether allicin induced the *I*_SC_ response by facilitating Na^+^ absorption via ENaC. Notably, the ENaC blocker amiloride (100 μM) had no significant effect on allicin-induced Δ*I*_SC_ (Figure 2B), ruling out the participation of Na^+^ transport in allicin-induced *I*_SC_ response. We then investigated whether anion secretion was involved in allicin-induced Δ*I*_SC_. Removal of ambient HCO_3_^−^ failed to affect the allicin-induced Δ*I*_SC_ (Figure 2D), while the *I*_SC_ response was significantly reduced in the solution without Cl^−^ (Figure 2C). Furthermore, when extracellular Cl^−^ and HCO_3_^−^ were both removed, the allicin-induced Δ*I*_SC_ was abolished (Figure 2E). The above results demonstrated that the allicin-induced *I*_SC_ response in airway epithelial cells was primarily Cl^−^ secretion.

### Involvement of CFTR in Allicin-Induced Cl^−^ Secretion

**FIGURE 3:**
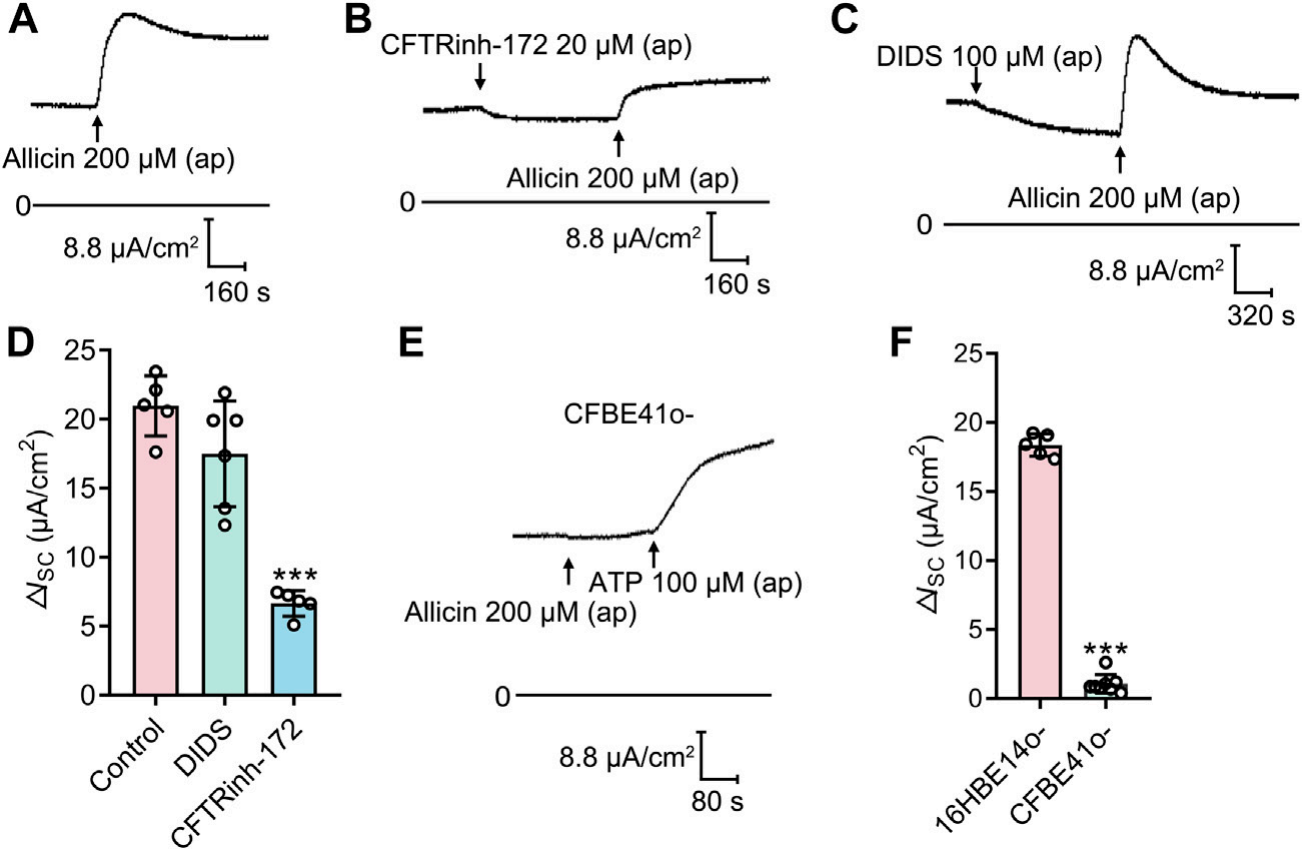
The short-circuit current (*I*_SC_) response stimulated by allicin was mediated by cystic fibrosis transmembrane conductance regulator (CFTR). **(A–C)** Representative traces of the *I*_SC_ responses induced by the allicin (200 μM) **(A)** without or with pretreatment of **(B)** CFTRinh-172 (20 μM) or **(C)** DIDS (100 μM) in 16HBE14o- cells, with **(D)** the corresponding statistical analysis. **(E)** Representative trace of the *I*_SC_ responses induced by the allicin in CFBE41o- cells, with **(F)** the corresponding statistical analysis. Each column and error bar indicate the mean ± SD. ^***^ *p* < 0.001 compared with the control or 16HBE14o- group.

To verify which Cl^−^ channel is responsible for allicin*-*induced *I*_SC_ response, several Cl^−^ channel blockers were used. Application of the CFTR blocker CFTRinh-172 (20 μM), but not the Ca^2+^ activated Cl^−^ channel (CaCC) blocker DIDS (100 μM), significantly attenuated the allicin-induced Δ*I*_SC_ (Figures 3A–D), which revealed the involvement of CFTR in this process. We further performed the experiment in a CF cell line, CFBE41o-, which is homozygous for the most common CF mutation, the deletion of phenylalanine at position 508 (Bruscia et al., 2002). The allicin-induced Δ*I*_SC_ was abrogated in CFBE41o- cells, whereas the CaCC-mediated Cl^−^ secretion elicited by ATP (100 μM) remained detectable (Figures 3E, F). These observations demonstrated that allicin-induced Cl^−^ secretion was mediated by CFTR.

### Involvement of cAMP Signaling in Allicin-Induced Cl^−^ Secretion

**FIGURE 4:**
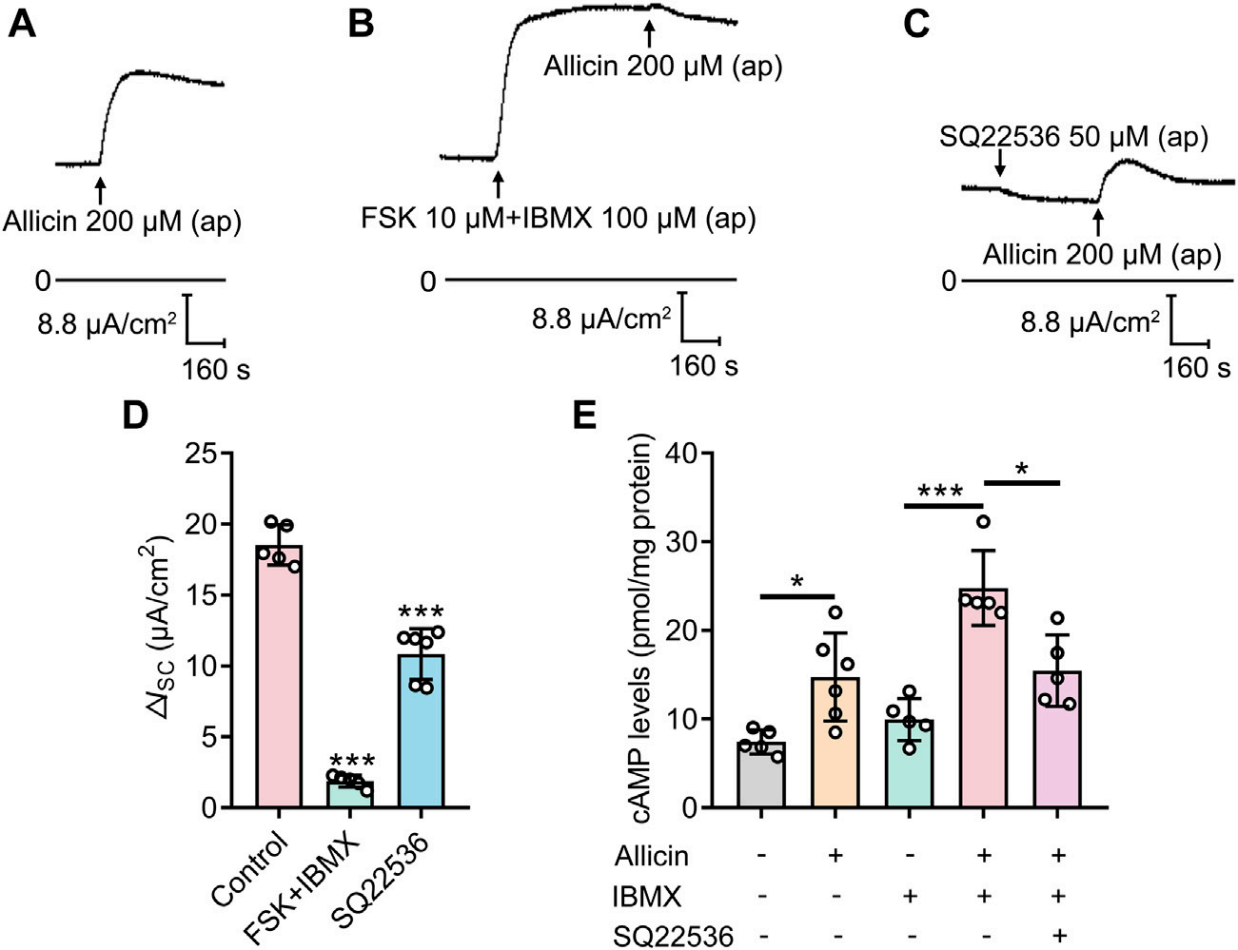
The short-circuit current (*I*_SC_) response stimulated by allicin was mediated by adenylate cyclase-cAMP signaling pathways. **(A–C)** Representative traces of the *I*_SC_ responses induced by the allicin (200 μM) **(A)** without or with pretreatment of **(B)** forskolin (FSK, 10 μM) and IBMX (100 μM), or **(C)** SQ22536 (50 μM) in 16HBE14o- cells, with **(D)** the corresponding statistical analysis. **(E)** Statistical analysis showing the effect of IBMX (100 μM) or SQ22536 (50 μM) on intracellular cAMP concentration after pretreatment with allicin (200 μM) in 16HBE14o- cells. Each column and error bar indicate the mean ± SD. ^*^ *p* < 0.05, ^***^ *p* < 0.001 compared with the control.

It is well known that the opening of CFTR is modulated by intracellular cAMP (Csanády et al., 2019). An increase in cAMP level can either be the result of the activation of adenylate cyclase (AC) or inhibition of phosphodiesterase (PDE) and vice versa. To verify whether allicin activated CFTR by increasing the intracellular cAMP level, FSK (10 μM), an AC activator, was used to stimulate the maximum AC activity while IBMX (100 μM), an inhibitor of PDE, was used to inhibit the degradation of cAMP. Pretreatment with FSK and IBMX abolished the *I*_SC_ response elicited by allicin (Figures 4B, D). Furthermore, the allicin-induced *I*_SC_ was significantly reduced by pretreatment of SQ22536 (50 μM), an inhibitor of AC (Figures 4C, D). Consistent with the *I*_SC_ results, the application of allicin triggered an elevation in intracellular cAMP level in an SQ22536-sensitive way (Figure 4E). The above results suggest that allicin modulates the activity of CFTR by increasing intracellular cAMP concentration via activation of AC activity rather than inhibition of PDE activity.

### Allicin Stimulated ASL Hydration *in vivo*

**FIGURE 5:**
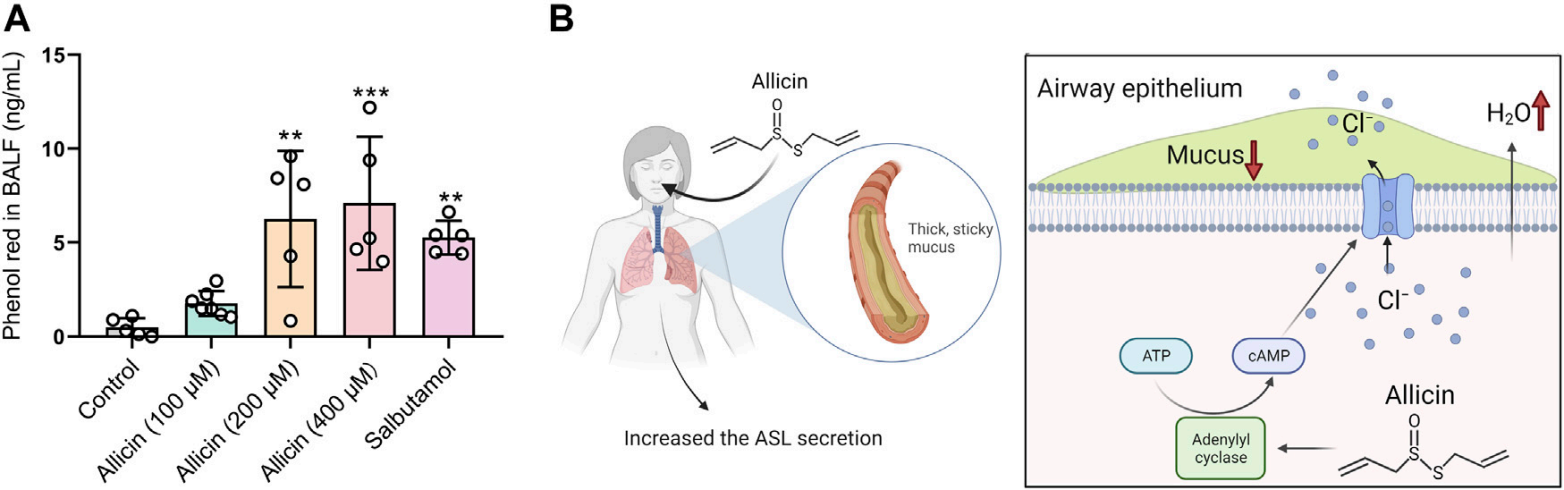
Allicin promoted tracheobronchial secretion in mice and the schematic diagram. **(A)** Statistical analysis showing the quantification of phenol red in bronchoalveolar fluid (BALF) of mice after administration of allicin (100 μM, 200 μM, 400 μM) or salbutamol (1 mg/ml). Each column and error bar indicate the mean ± SD. ^**^ *p* < 0.01, ^***^ *p* < 0.001 compared with the control. **(B)** Schematic drawing of allicin-induced ASL hydration. Allicin activated adenylate cyclase to increase the intracellular cAMP level in airway epithelial cells, which promoted the activation of cystic fibrosis transmembrane conductance regulator (CFTR) and transepithelial Cl^− ^as well as airway surface liquid (ASL) secretion. Inhalation of allicin may relieve symptoms of mucus defect-associated pulmonary diseases.

Transepithelial Cl^−^ secretion drives the water across the airway epithelium, leading to the hydration of the airway surface, which reflects the pharmacology of expectorant drugs. To investigate whether allicin harbors the mucosecretolytic activity by promoting ASL secretion, we evaluated the pro-secretory effect of allicin in murine models using the phenol red secretion assay. Similar to the mucokinetic agent salbutamol (also known as albuterol) (Menezes et al., 2019) (Rogers, 2007), inhaled allicin significantly increased the concentration of phenol red in BALF in a concentration-dependent manner (Figure 5A). These results indicated that allicin could facilitate ASL hydration by stimulating transepithelial Cl^−^ secretions *in vivo*.

## Discussion

As the major component from garlic, allicin has been shown to possess a variety of beneficial pharmacological and therapeutic effects, such as antimicrobial activity (Tsuchiya and Kawamata, 2019) (Fujisawa et al., 2009) and regulation of ion channel function (Macpherson et al., 2005). Here in our study, we demonstrated that allicin could activate AC to increase the intracellular cAMP level in airway epithelial cells, which resulted in CFTR activation and transepithelial Cl^−^ secretion. Furthermore, allicin showed mucosecretolytic activity by promoting ASL secretion in a mouse model (Figure 5B).

16HBE14o-, a simian virus 40-transformed human bronchial epithelial cell line, has been shown to express functional tight junction proteins and CFTR channel. The 16HBE14o-monolayers generate high transepithelial resistance and retain active Cl^−^ transport activity (Cozens et al., 1994), making it widely used as a suitable cell model for studying transepithelial Cl^−^ transport *in vitro*. Under physiological conditions, the apical membrane surface of airway epithelial cells is in contact with ASL with a low Cl^−^ concentration while the basal membrane surface is exposed to tissue fluid with a high Cl^−^ concentration. In our study, we simulated the physiological conditions for airway epithelium by establishing a basal-to-apical Cl^−^ gradient, which generated a mean PD of −6.55 ± 0.85 mV (*n* = 17), consistent with what has been previously described (Callaghan et al., 2020). Using this *in vitro* model, we demonstrated that allicin elicited CFTR activation and transepithelial Cl^−^ secretion. Consistent with these findings, a recent research reported that allicin increased the transepithelial PD of colon tissues in rats, which may be related to the secretion of Cl^−^ and HCO_3_^−^ (Tsuchiya and Kawamata, 2019). This evidence indicated that allicin-induced activation of CFTR and anion secretion may have far-reaching implications beyond the respiratory system.

In our study, we demonstrated that allicin stimulated the generation of cAMP by activating AC rather than inhibiting PDE in airway epithelial cells (Figure 4E). Previous studies have shown that cAMP generation could be promoted by allicin, which was significantly inhibited by applying propargylglycine, an inhibitor of the H_2_S-generating enzyme cystathionine γ lyase, in rat mesenteric arterial rings (Cui et al., 2020). This phenomenon indicated that allicin might activate the AC by producing endogenous H_2_S which reportedly stimulated AC activity in airway epithelial cells (Zhang et al., 2018). On the other hand, allicin has been shown to modify conserved redox-sensitive cysteine residues in abundant cellular proteins (Loi et al., 2019) (Chi et al., 2019) (Ogawa et al., 2016). Considering that cysteine 1,004 in AC6 served as the possible residues to regulate AC activity (Jaggupilli et al., 2018), allicin may also directly activate AC via oxidative modification of cysteine residues. Further research is required to investigate the mechanism underlying allicin-induced AC activation. Interestingly, our result revealed that apical application of allicin induced a significantly larger *I*_SC_ responses than basolateral application (Figures 1A–C). Given that the cysteine residues in nucleotide binding domains (Harrington and Kopito, 2002) and cysteine 343 in transmembrane segment 6 (Holstead et al., 2011) of CFTR are known to play key roles in gating the anion conduction path, we thus speculated that besides activating CFTR indirectly by elevating intracellular cAMP levels, allicin may also interact with CFTR protein, leading to the improved efficiency of CFTR Cl^−^ transport process. Further studies are needed to validate this hypothesis.

Currently, the treatment of airway mucus stasis is restricted to controlling symptoms (Laselva et al., 2021). It is urged to find safe and low-cost alternative therapies for airway mucus hypersecretion. Our *in vivo* study demonstrated that allicin promoted ASL liquid secretion in a murine model, indicating that allicin may be a promising secretory and expectorant agent for the treatment of ASL dehydration and mucus defect-associated pulmonary diseases, such as COPD or bronchiectasis. Research on the pharmacological effects of allicin has been ongoing since its discovery and isolation in 1944 and its molecular structure analyzed in 1948. The researchers detailed the synthesis and breakdown of allicin into a range of sulfur-containing compounds, and elucidated its effects on protein structure and cellular functions (Almatroodi et al., 2019). Allicin has been proven to confer the property of bacteria killing and relieve inflammation in the lungs (Bjarnsholt et al., 2005). However, in patients with cystic fibrosis, the *Pseudomonas aeruginosa* forms biofilms in the lung, which counteracted the therapeutic effect of allicin capsules (Smyth et al., 2010). Additionally, because allicin reacts readily with glutathione (GSH) when it entering the bloodstream, it is difficult to achieve a therapeutically relevant concentration of allicin in cells anywhere in the body via oral administration. Inhalable therapeutics have been shown to reduce systemic effects and improve delivery to the airway epithelium (Labiris and Dolovich, 2003) (Rau, 2005). In our experiment, allicin was administered by aerosol inhalation and showed mucosecretolytic activity. Thus, the inhaled approach might be an effective novel treatment strategy that could be developed under appropriate circumstances to achieve therapeutically effective concentrations of allicin vapor in the lungs (Reiter et al., 2017) (Reiter et al., 2019). It should be noted that allicin is membrane-permeable and reportedly induced apoptosis as well as inhibited cell proliferation in mammalian cells at sublethal doses (Gruhlke et al., 2019). These findings raise concerns regarding the balance between the effectiveness and toxicity of allicin. Moreover, in light of the low specificity in targeting the cAMP signaling pathways which is involved in a wide range of physiological responses, improvements in efficient and precise delivery of allicin might be conducive to minimizing the off-target side effects.

In conclusion, we demonstrated that allicin induced transepithelial Cl^−^ and liquid secretion across airway epithelium via activation of CFTR. Our study expanded the physiological function of allicin in the respiratory system and provided novel insights into the regulatory effect of allicin on Cl^−^ channels for a thorough understanding of allicin biology.

## Data Availability

The original contributions presented in the study are included in the article/supplementary material further inquiries can be directed to the corresponding authors.
